# Crystallographically Determined Etching and Its Relevance to the Metal-Assisted Catalytic Etching (MACE) of Silicon Powders

**DOI:** 10.3389/fchem.2018.00651

**Published:** 2019-01-07

**Authors:** Kurt W. Kolasinski, Bret A. Unger, Alexis T. Ernst, Mark Aindow

**Affiliations:** ^1^Department of Chemistry, West Chester University, West Chester, PA, United States; ^2^Department of Materials Science and Engineering, Institute of Materials Science, University of Connecticut, Storrs, CT, United States

**Keywords:** porous silicon, silicon nanowires, metallurgical grade silicon, etching, metal assisted catalytic etching, MACE, porous powder

## Abstract

Metal-assisted catalytic etching (MACE) using Ag nanoparticles as catalysts and H_2_O_2_ as oxidant has been performed on single-crystal Si wafers, single-crystal electronics grade Si powders, and polycrystalline metallurgical grade Si powders. The temperature dependence of the etch kinetics has been measured over the range 5–37°C. Etching is found to proceed preferentially in a 〈001〉 direction with an activation energy of ~0.4 eV on substrates with (001), (110), and (111) orientations. A quantitative model to explain the preference for etching in the 〈001〉 direction is developed and found to be consistent with the measured activation energies. Etching of metallurgical grade powders produces particles, the surfaces of which are covered primarily with porous silicon (por-Si) in the form of interconnected ridges. Silicon nanowires (SiNW) and bundles of SiNW can be harvested from these porous particles by ultrasonic agitation. Analysis of the forces acting between the metal nanoparticle catalyst and the Si particle demonstrates that strongly attractive electrostatic and van der Waals interactions ensure that the metal nanoparticles remain in intimate contact with the Si particles throughout the etch process. These attractive forces draw the catalyst toward the interior of the particle and explain why the powder particles are etched equivalently on all the exposed faces.

## Introduction

Silicon is poised to extend its range of application from primarily electronics and photovoltaics into drug delivery and energy storage. Nanostructured silicon has attracted significant interest for targeted delivery of multiple compounds in a theranostic setting (Salonen et al., 2008; Santos et al., 2011; Santos and Hirvonen, 2012). Porous silicon (por-Si) particles have been studied intensively for sustained release of drugs and used successfully to carry a wide variety of payloads from small-molecule drugs to therapeutic biomolecules, such as peptides, siRNA and DNA (Kaukonen et al., 2007; Anglin et al., 2008; Kilpeläinen et al., 2009; Ashley et al., 2011) as well as genes (Wareing et al., 2017). Nanostructured Si plays an increasingly important role in energy conversion and storage devices (Aricò et al., 2005; Kamat, 2007; Hochbaum and Yang, 2010; Micheli et al., 2013; Han et al., 2014; Mai et al., 2014).

Silicon nanowires (SiNW), and methods to produce them in industrial scale bulk quantities, are of particular interest in the realm of rechargeable lithium ion batteries (LIB). LIB have for all practical purposes reached the theoretical capacity of 372 mA h g^−1^ with respect to their graphitic anodes (Lee et al., 2016). Silicon has the greatest specific capacity (3,579 mA h g^−1^) among elements that alloy with lithium; thus, it is particularly attractive for advanced battery designs (Kasavajjula et al., 2007; Bruce et al., 2008; Mai et al., 2014; Lee et al., 2016) and its introduction into commercial batteries has begun (Blomgren, 2017). However, the nearly 400% volume expansion of Si upon full lithiation destroys bulk Si anodes. Nanostructuring of Si anodes can alleviate pulverization, which increases dramatically the reversibility of lithiation/delithiation cycles (Aricò et al., 2005; Shin et al., 2005; Kang et al., 2008; Kim et al., 2008; Leisner et al., 2010; Han et al., 2014). Silicon pillars (as SiNW are sometimes referred to in this field) are of particular interest for LIBs (Chan et al., 2008; Armstrong et al., 2014) because crystalline pillars with a cross section below 150 nm (Liu et al., 2012) and amorphous pillars with a cross section below 870 nm (McSweeney et al., 2015) retain their structural integrity upon cycling. The cycling behavior of SiNW is improved by porosification (McSweeney et al., 2015).

The crystallinity, and whether preferential crystallographic orientation of SiNW can be controlled, is not merely of academic interest, but is also potentially important for applications. Swelling of Si upon lithiation is strongly dependent on crystallographic orientation (Lee et al., 2011), expanding preferentially in the 〈110〉 directions (Liu et al., 2012). Thus, SiNW with sidewalls terminated by {110} planes will be particularly well-suited to lithiation/delithiation cycling with favorable kinetics and limited pulverization. SiNW with selected orientation along their long axis may be of interest for electronic devices since a significant enhancement of hole and electron mobilities was observed in 〈110〉-oriented SiNW compared to 〈001〉-oriented SiNWs with comparable diameters (Huang et al., 2009).

Metal-assisted catalytic etching (MACE) is a widely-used (Li, 2012; Han et al., 2014) method to produce either por-Si or SiNW. MACE takes advantage of the inherently faster kinetics of electron transfer at electrolyte/metal interfaces compared to semiconductor interfaces to catalyze etching of a semiconductor in the vicinity of a metal nanoparticle or patterned metal film deposited on the semiconductor surface (Li, 2012). Both local and remote etching can take place depending on reaction conditions (Chartier et al., 2008; Chiappini et al., 2010). Many aspects of the mechanism of catalysis remain unresolved because of uncertainties in the electronic structure of the metal/semiconductor interface and its role in electron transfer (Kolasinski, 2014, 2016). The vertical direction and its relationship to crystallographic axes are defined readily during the etching of flat single-crystal wafers. However, when etching powders, there is no obvious vertical direction because the particles not only exhibit roughness and irregularity of shape, but also they may be polycrystalline. A question that arises naturally is whether this difference will cause any differences in the structures that are etched in powders relative to wafers.

Pore formation by anodic etching is known to exhibit some degree of crystallographic preference. As demonstrated by Föll et al. (2002) a variety of pore geometries are accessible. Key parameters for determining the pore morphology are the electrolyte type, e.g., whether it is aqueous vs. organic or possibly oxidizing, the HF concentration, doping level and type, and in some cases the illumination state (front side vs. back side). With macropores on n-type Si formed in an aqueous electrolyte with backside illumination, pores grow exclusively in 〈001〉 directions and (occasionally, if all available 〈001〉 directions are inclined steeply) in 〈113〉 directions. Their morphology is always describable as a main pore in one of these two directions and side pores or branches in some of the others. Mesopores with diameters 10 nm ≤ *d* ≤ 50 nm grow in 〈001〉 directions and branch at right angles to these into other 〈001〉 directions. However, at high current densities the geometrical shape of the pore walls is lost while the direction of the pore axis is still along a 〈001〉 direction.

The crystallographic orientations of etch track pores and SiNW produced by MACE were originally thought to be determined solely by the substrate crystallography (Peng et al., 2005). However, the dependence is more complex (Peng et al., 2008), and reports of the crystallographic dependence of MACE are often contradictory, perhaps because analysis by cross-sectional scanning electron microscopy (SEM) is difficult to interpret unambiguously with regard to directionality unless cross sectional cleavages are made in more than one known direction. Whereas etching on Si(001) wafers, even on wafers with significant miscut angles (Ma et al., 2013), is reported to proceed along 〈001〉 directions (Peng et al., 2007) even when the temperature is varied from 0–50°C (Cheng et al., 2008), the results on wafers of other orientations are much more varied.

It was initially reported (Huang et al., 2009) that [110]-oriented SiNW could not be obtained by electroless deposition of Ag on a Si(110) wafer, and that only a Ag film with a lithographically defined mesh of openings could be used to form [110]-oriented SiNW. This was later reported not to be the case (Huang et al., 2010). Nonetheless, a metal film with holes always preferentially catalyzes etching along the vertical direction of a wafer (more accurately normal to the wafer surface) even on Si(113) (Peng et al., 2007) and polycrystalline wafers (Toor et al., 2016b).

The concentration of the oxidant was shown by Huang et al. (2010) to be an important factor affecting the etching direction on non-(001) oriented substrates, e.g., both (111) and (110). On (110) substrates at *low oxidation concentrations*, etching along an inclined 〈001〉 direction was found. However, the preferred etching direction is along the normal [110] direction for *high oxidant concentration*. In metal-assisted anodic etching, the current density can be controlled to affect the same change in preferred etch direction, which facilitates the formation of zigzag orientation-modulated pores. Similar results were found for (111) substrates, which etch along the normal [111] direction at *high concentration* but along the 〈001〉 directions for *low concentration* regardless of whether the etchant is H_2_O_2_ or Fe(NO_3_)_3_.

The opposite behavior has been reported for Ag-catalyzed etching of Si(111) in H_2_O_2_ + HF. Pei et al. (2017) found that [111]-oriented nanowires are observed for 20 mM H_2_O_2_ but [001]-oriented SiNWs are formed at 400 mM H_2_O_2_. Ghosh and Giri (2016) similarly reported that the etch direction changed from the vertical 〈111〉 direction to slanted and eventually to wavy as H_2_O_2_ concentration is increased.

Temperature is also reported to be an important factor, increasing the rate of etching with an activation energy estimated to be 0.36 eV (Cheng et al., 2008) as well as affecting the direction of etching on non-(001) wafers. On Si(111) Pei et al. (2017) reported that [111]–directed etching is favored by low *T* and [001]-directed etching by high *T*. The higher the concentration of H_2_O_2_, the lower the transition temperature from [111] to [001]. On the other hand, Bai et al. (2013) reported that for etching of Si(111) with AgNO_3_ + HF solution, the etch direction could be switched from 〈112〉 at 10°C to 〈113〉 at 20°C to 〈111〉 above 30°C.

Below we investigate the etch direction on three types of substrates: flat single crystal wafers, single-crystal wafers textured with crystallographically-defined macropores, and silicon powder (both polycrystalline metallurgical grade and single-crystal wafer reclaim). Crystallographically-defined macropores are produced by methods that have been described previously (Mills and Kolasinski, 2005; Dudley and Kolasinski, 2008). These samples allow us to prepare bulk single-crystals that present simultaneously several well-defined surfaces with different orientations. Here we report on the crystallographic dependence of MACE and develop a model that addresses quantitatively aspects of this dependence. With the aid of this model, and analysis of the forces acting between the metal nanoparticle and the silicon substrate, we explain why the etching of powders can lead to similar etch-track-pore structures as those found on wafers.

## Experimental

### Laser Ablation and Macropore Formation

Si wafers (University Wafers: Si(001) prime grade, 0–100 Ω cm, B doped, p type; Si(110) prime grade 1–10 Ω cm, B doped, p type; Si(111) mechanical grade, unspecified doping) with 500 μm thickness were ablated using a Spectra-Physics Quanta Ray INDI-HG-205 Nd:YAG laser producing radiation with 355 nm or 532 nm wavelengths, 6 ns pulsewidths, and 115–175 mJ pulse energies. Adjacent stripes (1.25–2.5 mm spacing) were irradiated along the Si wafers by translating the ablation stage with 0.04–0.16 mm s^−1^ scan rate. The beam was focused softly by placing the sample ~30–35 cm in front of the focal point of a *f* = 50 cm lens. The pressure of 5% SF_6_ in N_2_ (Praxair) was maintained in the range of 1–10 kPa in the ablation chamber. Pure N_2_ or Ar can also be used, though these tend to make blunter pillars and less well-defined macropores. Prior to ablation, Si wafers were cleaned by sonication for 5 min in acetone and 5 min in ethanol. After ablation, wafers were etched chemically to form crystallographically-defined macropores by immersion for 100–140 s in 40% KOH(aq) solution held at 80°C (VWR ACS reagent grade). After chemical etching, samples are rinsed in 0.2 M HCl (Fisher ACS certified), deionized (DI) H_2_O, and ethanol (Pharmco-Aaper anhydrous ACS/USP grade), then dried with a stream of Ar.

### Metal Assisted Catalytic Etching of Wafers

MACE was performed with Ag nanoparticles deposited at a low enough density that they should be able to etch as individual particles, rather than as a continuous film. Wafers were placed in 4 mL HF (Acros Organics 49% ACS reagent) in separate containers. To the wafers was added a separate solution of 3 drops 50.4 mM AgNO_3_ (Fisher ACS reagent), 2 mL concentrated acetic acid (Fisher ACS reagent), and 2 mL deionized (DI) H_2_O. After 10 min, the wafers were transferred to a mixture of 5 mL concentrated HF, 2 mL acetic acid, and 3 mL DI H_2_O. To this container was added a solution of 0.2 mL 35% H_2_O_2_ (Acros Organics 35% ACS reagent), 2.5 mL conc. HF, and 2.5 mL DI H_2_O. The wafers were etched for 4 min, rinsed in DI H_2_O and ethanol, and dried with Ar. The etchant is 0.15 M H_2_O_2_.

### Metal Assisted Catalytic Etching of Powders

Etching of powders is performed using either polycrystalline metallurgical-grade particles from Elkem Silicon Materials or unpolished single crystal reclaimed wafer chunks from Dow Chemical. H_2_O_2_, HNO_3_, FeCl_3_•6H_2_O, Fe(NO_3_)_3_, and V_2_O_5_ have all been used as the oxidant but all kinetics data were obtained using H_2_O_2_. Addition of oxidant can either be made all at the beginning of the etch cycle or at a steady rate with a syringe pump. Just as in regenerative electroless etching (ReEtching) (Kolasinski et al., 2017), addition of oxidant with a syringe pump leads to a more controlled etching process with improved thermal management, a steadier rate of etching, improved yield, and most importantly independent control of the rate and extent of etching (Kolasinski et al., 2018). To ~0.1 g Si is added 17.5 mL concentrated HF, 10 mL DI H_2_O, 2.5 mL acetic acid, and 20 mL 0.06 M AgNO_3_. After 10 min the contents are decanted and 17.5 mL concentrated HF, 18 mL DI H_2_O, and 12.5 mL Fe(NO_3_)_3_ are added to the Si. After 1–2 min of etching, 2 mL 0.06 M AgNO_3_ is added. The Si etches for 15 min with stirring, and the contents are decanted. A 1:1 mixture of HNO_3_ (Fisher ACS reagent) and H_2_O is used to dissolve Ag. The Si is rinsed with DI water and pentane (Alfa Aesar environmental grade 98+%), then dried in an evacuated desiccator.

A different etch procedure, performed at 0°C with slow addition of metal catalyst and oxidant, produces por-Si/SiNWs of different quality. This method allows for control of porous film morphology by varying the concentration of oxidant injected. To 0.1 g Si is added 17.5 mL conc. HF, 10 mL DI H_2_O, 2.5 mL acetic acid, and 20 mL 0.06 M AgNO_3_. The AgNO_3_ is added over the course of 8 min, but nucleation occurs for an additional 6 min before the contents are decanted. To the Si is added 30 mL DI H_2_O and 17.5 mL concentrated HF. About 0.65 mL 6% H_2_O_2_ is injected into the solution over 16 min, and an additional 2 mL 0.06 M AgNO_3_ is added slowly to the container after about 5 min etching time. The contents are decanted, and the Si is rinsed with a 1:1 mixture of HNO_3_ and H_2_O as well as 0.2 M HCl, DI water and pentane. The Si is dried in an evacuated desiccator.

### Electron Microscopy Sample Preparation

Microstructural data were collected using a combination of advanced electron microscopy techniques. Secondary electron (SE) SEM images were acquired from MAC-etched shards of metallurgical and single-crystal electronics grade Si using an FEI Verios 460L SEM operating at an accelerating voltage of 2 kV. MAC-etched Si wafers were examined in an FEI Teneo LVSEM using an accelerating voltage of 5 kV. SE SEM images were acquired from cleaved samples to reveal the macropore geometry in cross-section. Cross-sectional TEM samples were produced from the MAC-etched silicon wafers using focused ion beam (FIB) techniques in an FEI Helios NanoLab 460F1 dual-beam FIB-SEM. TEM lamellae were prepared from the macropore by depositing a Pt layer *in-situ* to protect the near-surface region during Ga^+^ ion milling. Parallel trenches were then milled on either side of the Pt strap to define a pre-thinned lamella. The lamella was then transferred to a Cu Omni grid using a micro-manipulator needle; final thinning was performed at 30 kV. The FIB lift-outs were then analyzed in an FEI Talos F200X scanning transmission electron microscopy (STEM) operating at an accelerating voltage of 200 kV.

## Results

### Etching of Powders

**Figure 1:**
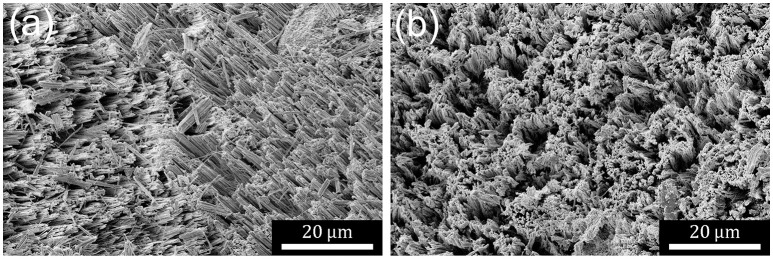
**(a)** SE SEM micrograph showing a typical metallurgical grade powder particle after MACE. Etch track pores form structures in domains with different axial directions. Particles are porosified to expose several different morphologies. **(b)** SE SEM micrograph showing a typical single-crystal electronics grade powder particle after MACE. Domain sizes are much greater and the angle of the etching axis deviates less from the local normal than observed for metallurgical grade Si.

MACE has been performed on Si powder, for which there is no unique upward, vertical or normal direction. As shown in Figure 1, domains of etch-track pores form that tend to point in the same direction within local domains. Some regions have the appearance of nanowires or blade-like ridges whereas other regions maintain the original flatness of the particle and etch porous regions with interconnected pore walls. Single-crystal powder particles tend to exhibit a single domain on each particle face with the axis of the etch track pores along a single direction. This can also occur on polycrystalline powder particles, particularly smaller ones. More typically, polycrystalline particles exhibit domains with etch axes that point in multiple directions with respect to the local surface normal. In some cases, structures on the same metallurgical-grade particle face can even be perpendicular to one another. The pores often exhibit a wavy character on single-crystal particles and are usually straighter on polycrystalline particles.

It has long been known that porous silicon films can be pulverized to form microparticles (Heinrich et al., 1992). Similarly it is known that capillary forces acting either during etching or drying (Campbell et al., 1995; Bellet and Canham, 1998) can change the structure of highly porous films. MAC-etched porosified particles are no different in this respect to MAC-etched porosified wafers. The ridge-like structures can be harvested from MAC-etched Si particles to produce individual SiNW and bundles of SiNW with lengths of several or even tens of micrometers.

### Etching of Macropores

**Figure 2:**
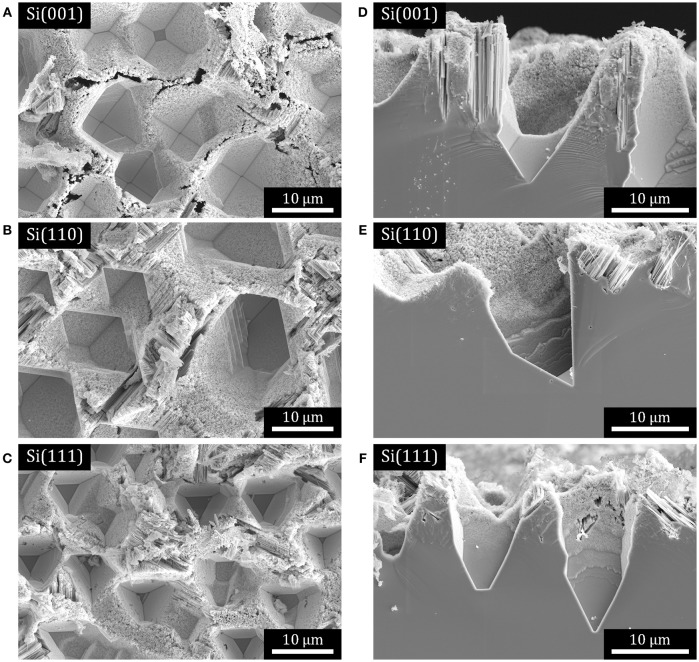
SE SEM micrographs of macropores on Si substrates that have been subjected to Ag-catalyzed metal assisted etching in H_2_O_2_ + HF solution. **(A–C)** Plan view micrographs of: **(A)** rectangular macropores on Si(001), **(B)** parallelepiped macropores on Si(110), and **(C)** triangular macropores on Si(111). **(D–F)** cross-sections of pores in the samples shown in **(A–C)**, respectively.

As previously reported (Mills et al., 2005; Dudley and Kolasinski, 2008), anisotropic KOH etching of laser ablation pillars leads to the formation of rectangular macropores on Si(001) substrates. On Si(111) substrates, macropores are initially hexagonal then progressively become triangular in shape. These results are confirmed in Figures 2A,C. Here we show, Figure 2B, that in addition parallelepiped macropores are formed when laser ablation pillars formed on Si(110) substrates are etched anisotropically.

The macropores represent single-crystal substrates with well-defined bulk orientations that also exhibit a variety of surface facets with well-defined but nonetheless very different orientations from the bulk orientation, as shown in the cross-sectional images in Figures 2D–F. Performing MACE on these substrates allows us to consider the influence of bulk orientation, surface orientation and, thus, whether etching along a local surface normal is different from etching along the normal to the macroscopic wafer surface. Figure 2 demonstrates that most if not all exposed facets are porosified by the formation of etch track pores.

**Figure 3:**
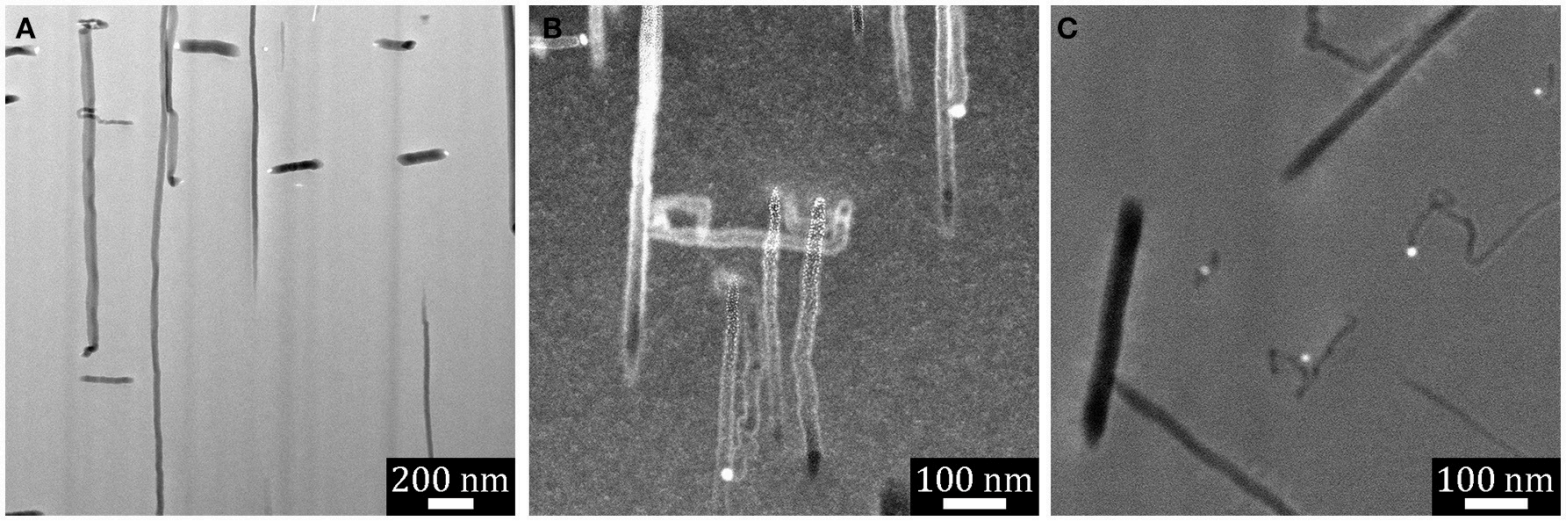
HAADF STEM images of FIB-cut cross-sections taken from the walls of macropores on Si substrates that had been subjected to Ag-catalyzed metal assisted etching in H_2_O_2_ + HF solution: **(A)** Si(001), **(B)** Si(110), **(C)** Si(111).

MAC-etched macropores in substrates with all three orientations were cross-sectioned by FIB to form electron-transparent specimens suitable for imaging with STEM. Three representative high-angle annular dark-field (HAADF) STEM images are displayed in Figure 3. For all three substrates the primary direction of etching is found along the 〈001〉 directions, with relatively few turns observed and only the occasional isolated meandering etch track. In each case, there are Ag nanoparticles found at the end of some of the etch track pores in these specimens. The Ag nanoparticles appear bright due to strong Z contrast in the HAADF images. We note that these nanoparticles were observed far more frequently than one would expect based upon random sampling of the etch track structure. The proximity of the Ag nanoparticles to one another is clear evidence that the MAC etching must involve some co-operative process.

In-depth analysis of the microstructure of MAC-etched wafers and particles will be presented elsewhere. Here we concentrate on the crystallographic dependence of the etch track pores. We note also that SEM images of MAC-etched wafers and particles often look as though SiNWs have been formed. Indeed, often a few SiNWs are observed at the edges of etched domains. However, these SiNWs are most likely the result of cleavage caused either by H_2_ bubble formation or during drying. The analysis of FIB cross sections reveals that the MAC-etched film is comprised predominantly of pores with interconnected walls rather than free-standing nanowires.

### Temperature Dependence of Etch Rate

**Figure 4:**
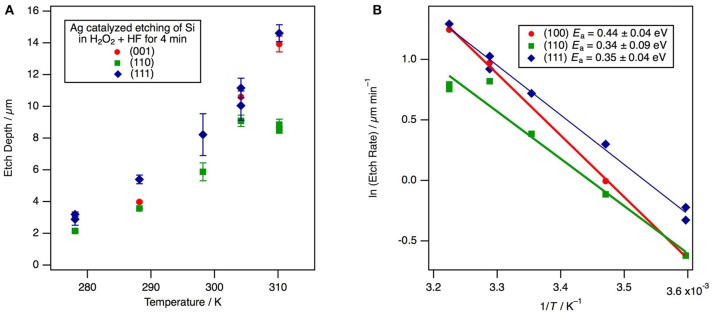
The temperature dependence for etching along the 〈001〉 direction of Ag-catalyzed etch rate for (001)-, (110)-, and (111)-oriented Si substrates in H_2_O_2_ + HF solutions. For all three substrate orientations the primary direction of etching is along the 〈001〉 directions. **(A)** A plot of the etch depth vs. temperature at a constant etch duration of 4 min. Each point represents the mean of multiple depth measurements from an individual experiment with error bars defined by one standard deviation. **(B)** An Arrhenius plot of the etch rate vs. inverse temperature.

If the oxidant concentration remains constant during etching, we expect the etch depth to increase linearly in time, an observation that has been confirmed experimentally (Ghosh and Giri, 2016; Toor et al., 2016a). If we assume Arrhenius behavior and that the kinetic order is independent of temperature, the etch rate is given by the depth along the etch direction divided by time, and a plot of the etch rate vs. inverse temperature should yield a straight line as long as the concentration of the oxidant is essentially invariant during the etch (as it is for wafer etching, but not necessarily for high-surface area powder etching). The results are shown in Figure 4 and the expected linear behavior indicative of an activation energy *E*_a_ = −*k*_B_(slope) is observed.

One measure of the uncertainty in the etch rate is obtained by averaging multiple measurements of the depth across the wafer as obtained from cross sectional SEM images. Generally this variance is less than the sample-to-sample variance between experiments run under identical conditions; hence, the reported experimental uncertainty likely underestimates the true uncertainty. Because etching is found to occur along 〈001〉 directions for all substrate orientations, one might expect the activation energy to be similar on each substrate orientation. The activation energy is found to be 0.44 ± 0.05 eV on Si(001), 0.34 ± 0.09 eV on Si(110), and 0.35 ± 0.05 eV on Si(111). The measured activation energies are insignificantly different on Si(110) and Si(111), but the larger value for Si(001) is on the border of statistically significance. Therefore, it appears that vertical as opposed to angled etching makes little, if any, difference to the mechanism of etching. These results are within the experimental uncertainty of the previously reported value of *E*_a_ = 0.36 eV (Cheng et al., 2008) for Si(001). The experimentally-determined activation energy is also similar in magnitude to the estimate of the etch energy of 0.31 eV from the model that will be developed below.

## Discussion

### Modeling of Forces Between the Ag Nanoparticle and the Si Substrate

**Figure 5:**
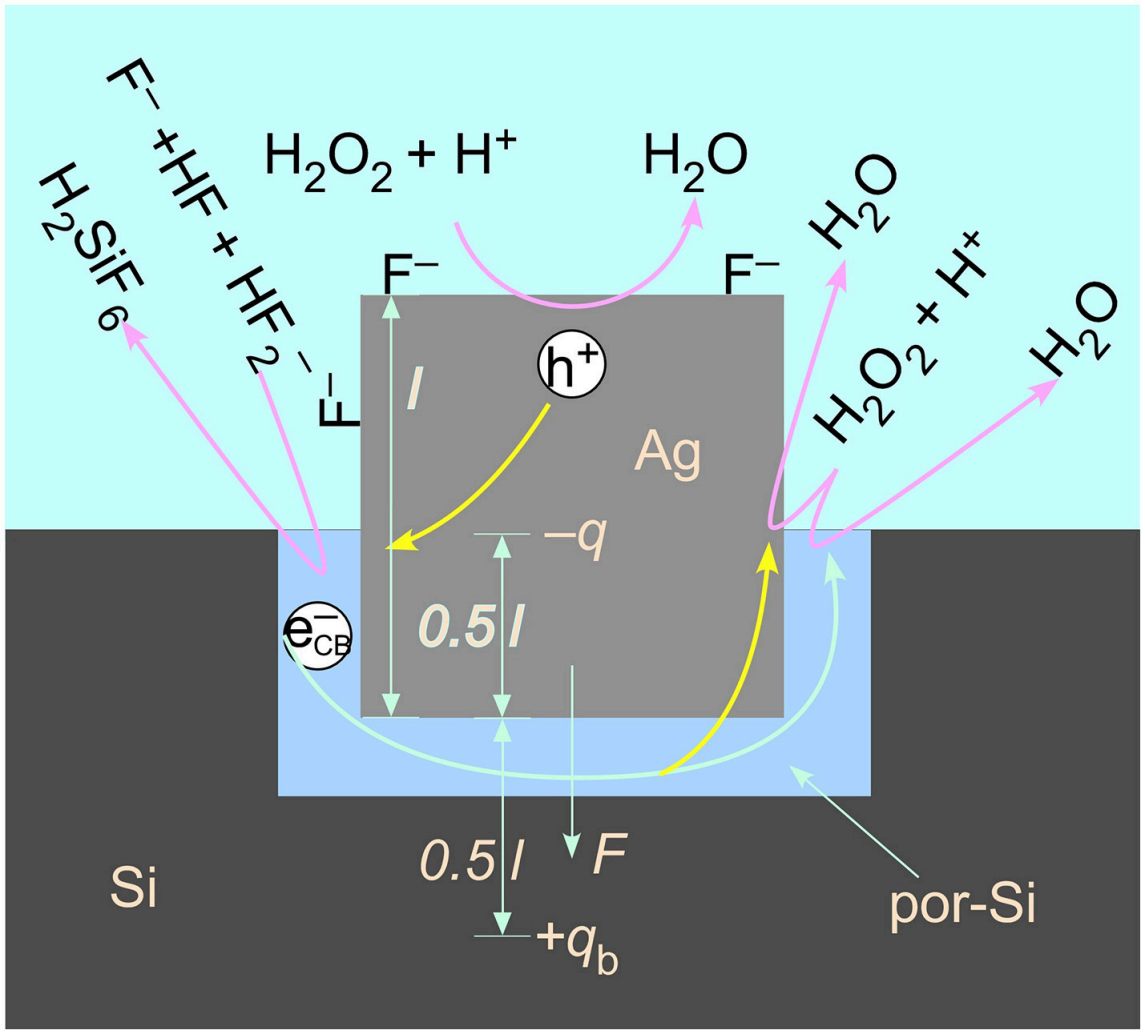
A model of the electrostatic interaction of a charged particle and its image charge located in bulk Si across an intervening porous region. An excess of adsorbed anions, such as F^−^, leads to a net negative charge on the metal catalyst. Reactions pertinent to valence band hole injection by H_2_O_2_ decomposition, Si etching by complexation of Si to H_2_SiF_6_ by fluoride species, and capture by H_2_O_2_ of the conduction band electron injected by F^−^ (which can occur either on the metal nanoparticle or on the Si) are also represented. The formation of a porous region in the immediate proximity of the metal/Si interface facilitates the transport of reactants into and products out of the reaction zone.

Figure 5 depicts schematically the chemical events that are occurring during MACE. H_2_O_2_ undergoes an electrochemical half-reaction that removes electrons from the Ag nanoparticle,

(1) $$\begin{array}{l} {\text{H}}_{2}{\text{O}}_{2}+2{\text{H}}^{+}+2{\text{e}}^{-}\to 2{\text{H}}_{2}\text{O},\\ \;E_{298\;\text{K}}^{{}^{{}^{\circ}}}=1.776\;\text{V},\;\Delta G_{298\;\text{K}}^{{}^{{}^{\circ}}}=-342.7\;\text{kJ }\text{mo}{\text{l}}^{-1}. \end{array}$$

A hole injected into a Ag nanoparticle of side length *l* is more stable in the nanoparticle than at either the interface or in the Si. There is a barrier (Kolasinski, 2014) to electron transfer from Ag to Si, and tunneling through this Schottky barrier has been implicated as the primary means of charge transfer between the metal and Si (Rezvani et al., 2016). Therefore, a steady-state electron imbalance builds up in the Ag nanoparticle. This effective positive charge is offset by the adsorption of anions such as F^−^, which leads to an overall negative charge on the metal catalyst, as demanded by the negative voltage measured during etching by Rezvani et al. (2016).

#### Electrostatic Force

The charge imbalance on the Ag catalyst is modeled as a negative charge –*q* at the center of the cubic nanoparticle. The charge polarizes the Si beneath it. The polarization is modeled as an image charge +*q*_b_ located a distance 0.5 *l* below the nanoparticle. The charges are related to one another by a ratio involving the susceptibility of Si (Griffiths, 1981),

(2) $$\begin{array}{r} q_{\text{b}}=\frac{\chi_{{}_{\text{e}}}}{\chi_{{}_{\text{e}}}+2}q. \end{array}$$

The susceptibility of Si is 10.9, thus *q*_b_ = 0.845*q*. It has been shown (Kolasinski et al., 2015) that the primary means of Si etching is a valence 2 process for catalysis by Ag and Au. That is, the primary means of Si atom removal is porous Si formation. Therefore, the effective voltage associated with the charge imbalance on the Ag nanoparticle must have a magnitude <2 V, otherwise the etching would switch to electropolishing, a valence 4 process. This sets an upper bound on the net charge *q* on the nanoparticle that is consistent with the potential differences measured by Rezvani et al. of −0.5 V for Au and −0.2 V for Ag.

The voltage at the interface of the metal particle with the Si is given by

(3) $$\begin{array}{r} V=\frac{1}{4\pi \varepsilon_{0}}\frac{q}{0.5l}. \end{array}$$

The force generated by the attraction of this charge to its image charge is

(4) $$\begin{array}{r} F=\frac{1}{4\pi \varepsilon_{0}}\frac{qq_{\text{b}}}{l^{2}}=\pi \varepsilon_{0}V^{2}\frac{\chi_{{}_{\text{e}}}}{\chi_{{}_{\text{e}}}+2}, \end{array}$$

which is independent of the length of the particle. Upon substitution with *V* = −0.2 V, the electrostatic attraction between the Ag nanoparticle and the Si substrate is found to be 9 × 10^−13^ N. For a sense of scale, a nanoparticle with *l* = 100 nm has approximately 10^9^ valence electrons. An excess of just 7 units of elementary charge is sufficient to engender an effective voltage of −0.2 V. The mass of this particle is 10^−17^ kg, which means that the electrostatic attraction is over 9,000 times larger than the force of gravity. Contrary to some assertions in the literature, the force of gravity is irrelevant for holding the catalyst in contact with the Si surface. The electrostatic force drives the nanoparticle toward the image charge and holds the catalyst tight against the Si surface irrespective of the orientation of the surface compared to the laboratory vertical direction.

Since pressure is force per unit area and the particles have a square contact with the Si, the effective pressure induced by the image force is

(5) $$\begin{array}{r} p=\frac{\pi \varepsilon_{0}{\;V}^{2}\chi_{{}_{\text{e}}}}{\chi_{{}_{\text{e}}}\;+\;2}\frac{1}{l^{2}}. \end{array}$$

Thus, unlike force, pressure does scale with particle size. This amounts to 9.4 kPa at *r* = 10 nm but only 94 Pa at 100 nm. As we show below, the relative importance of van der Waals forces to electrostatic forces increases as particle size increases.

#### van der Waals Interaction

van der Waals forces, more specifically called Derjaguin and Landau, Verwey and Overbeek (DLVO) interactions, are non-linear and operate over a short distance; small changes in the gap distance or gap locations between the metal and Si will result in large changes in these attractive forces. However, the electrostatic forces calculated above are much less sensitive to surface roughness and gap variations. The combination of these two sets of forces ensures that the metal catalyst is always attracted strongly to the Si substrate and never loses contact with it as long as the metal is not pinned. This combination also enables the etching of all facets of powder particles irrespective of how the particles are oriented in the solution because these forces act to draw the metal catalyst toward the core of the Si particle.

Lai et al. (2013) have found that MACE was capable of deforming a pinned metal catalyst with a length of 2 μm and a width of 315 nm. They estimated that that the porous region below this catalyst was 2–4 nm thick and that the roughness of the Au catalyst surface was 5 nm at the Au/Si interface. The pressure required to deform the catalyst was 1–3 MPa, which they attributed to van der Waals forces.

Wong et al. (Hildreth et al., 2011, 2013; Rykaczewski et al., 2011) have also used pinned metal catalysts to estimate the forces experienced during MACE. The Pt catalysts had a width of 1 μm. They unequivocally showed that the electrophoretic model of Peng et al. (2008) cannot generate the forces required to deform their catalysts. They suggested that DLVO interactions were responsible for the attractive force between the catalyst and the Si substrate. They measured forces of 0.55–3.5 μN corresponding to pressure of 0.5–3.9 MPa.

To calculate the magnitude of DVLO interactions (Israelachvili, 2011), we start with the Hamaker constant, which is given by

(6) $$\begin{array}{r} A=\pi^{2}C\rho_{1}\rho_{2} \end{array}$$

where *ρ*_1_ and *ρ*_2_ are the number densities of the two materials and *C* is the London dispersion constant

(7) $$\begin{array}{r} C=\frac{3\alpha_{0}^{2}I}{4{\left(4\pi \varepsilon_{0}\right)}^{2}} \end{array}$$

where *a*_0_ is the polarizability, *I* is the ionization energy and ϵ_0_ the vacuum permittivity. For the interaction of two flat surface separated by *D*, which we assume here to be the thickness of the porous region beneath the catalytic particle, the energy per unit area of interaction is

(8) $$\begin{array}{r} W_{\text{vdW}}=\frac{A}{12\pi {\;D}^{2}}, \end{array}$$

and the pressure is

(9) $$\begin{array}{r} p_{\text{vdW}}=\frac{A}{6\pi {\;D}^{3}}. \end{array}$$

At *D* = 3 nm, this amounts to *p*_vdW_ = 542 kPa independent of the particle size in terms of surface area. Therefore, since the pressure associated with the electrostatic force decreases with increasing particle size, as particle size increases, the relative importance of the van der Waals forces increases. On the other hand, whereas the electrostatic force is not affected by a change in the thickness of the sub-particle porous region, the van der Waals force depends strongly on this distance as *D*^3^. Consequently, *p*_vdW_ increases from 542 kPa at *D* = 3 nm to *p*_vdW_ = 3.9 MPa at *D* = 1.55 nm. The range 1.55 nm ≤ *D* ≤ 3 nm fits the range of pressures measured during MACE by Wong et al. (Hildreth et al., 2011, 2013; Rykaczewski et al., 2011). Clearly then, the net effect of the electrostatic and van der Waals forces is to adhere the Ag nanoparticle tightly to the Si particle surface at all stages throughout etching as long as the metal nanoparticle is not pinned by lithographic construction.

### Etch Model

A wide variety of oxidants has been used in metal-assisted etching including H_2_O_2_, V_2_O_5_, HNO_3_, Fe(NO_3_)_3_, and dissolved O_2_ among others. These species have different kinetics of hole injection leading to, for example, different concentration dependences. However, once a hole has been injected into the Si valence band by any oxidant, the hole rapidly relaxes to the valence band maximum to initiate etching in a manner that is completely independent of the chemical identity of the oxidant. Dissolved V_2_O_5_ in HF has the advantage of being readily detected by absorption spectroscopy in both its oxidized 5+ (VO${}_{2}^{+}$) and reduced 4+ (VO^2+^) forms (Kolasinski and Barclay, 2013). This allowed Kolasinski et al. (2015) to establish that MACE catalyzed by both Ag and Au follows a valence two process. This demonstrates that the primary means of Si atom removal is porous Si formation through the current doubling pathway of the Gerischer mechanism (Kolasinski, 2003) rather than electropolishing. Electropolishing is a valence four process that involves SiO_2_ formation followed by HF stripping of the oxide. This conclusion is strictly true for a low concentration (~20 mM) of VO${}_{2}^{+}$ in 5 M HF. It is possible that at high oxidant concentration, the primary Si atom removal mechanism switches either to the valence four current quadrupling pathway of the Gerischer mechanism or else to electropolishing.

The results of these V_2_O_5_ + HF experiments (Kolasinski et al., 2015) are consistent with the work of Chartier et al. (2008) who found that the structure of a Si substrate subjected to metal assisted etching depends on the oxidant concentration or, stated more precisely, the concentration ratio of oxidant to HF. At low oxidant concentration (i.e., high HF/H_2_O_2_ ratio) they found no formation of oxide at the surface and etching localized to the catalyst, which results in the formation of meso- and macro-pores depending on the Ag nanoparticle size. This corresponds to the solution composition in the SiNW formation regime. At high oxidant concentration (low HF/H_2_O_2_ ratio) the Si surface is oxidized, the injected holes are distributed homogeneously, and thus electropolishing occurs. Complete electropolishing is incompatible with etch track pore and SiNW formation. These results indicate clearly that etching beneath the metal catalyst depends on the oxidant concentration (and HF/H_2_O_2_ ratio). Such dependence can lead to changes in the surface chemistry (a change in the adsorbates covering the surface as well as the etch mechanism), which could possibly lead to changes in the crystallographic dependence of MACE. A dependence of etch track pore direction on oxidant concentration was suggested by Huang et al. (2010).

Cooperative effects between metal particles influence MACE. This is evident, as noted above, from the frequent observation of Ag nanoparticles within lamellae produced for STEM imaging. In addition, cooperative effects are evinced by the observation that helical pores only occur with isolated metal particles [preferentially Pt (Tsujino and Matsumura, 2005)] and by the remarkably uniform etch depth and direction both across a wafer and across powder particles. Possible mechanisms for co-ordination include image forces between the metal particles, and/or the influence of band bending at the metal/Si interface from neighboring metal particles, which influences carrier transfer through the metal/Si interface (Huang et al., 2009). Another possibility is the diffusion of a chemical species such as F^−^ into Si in the wake of etching. Incorporation of F^−^ has been observed by Rezvani et al. (2016) and could alter effective doping levels and band bending.

**Figure 6:**
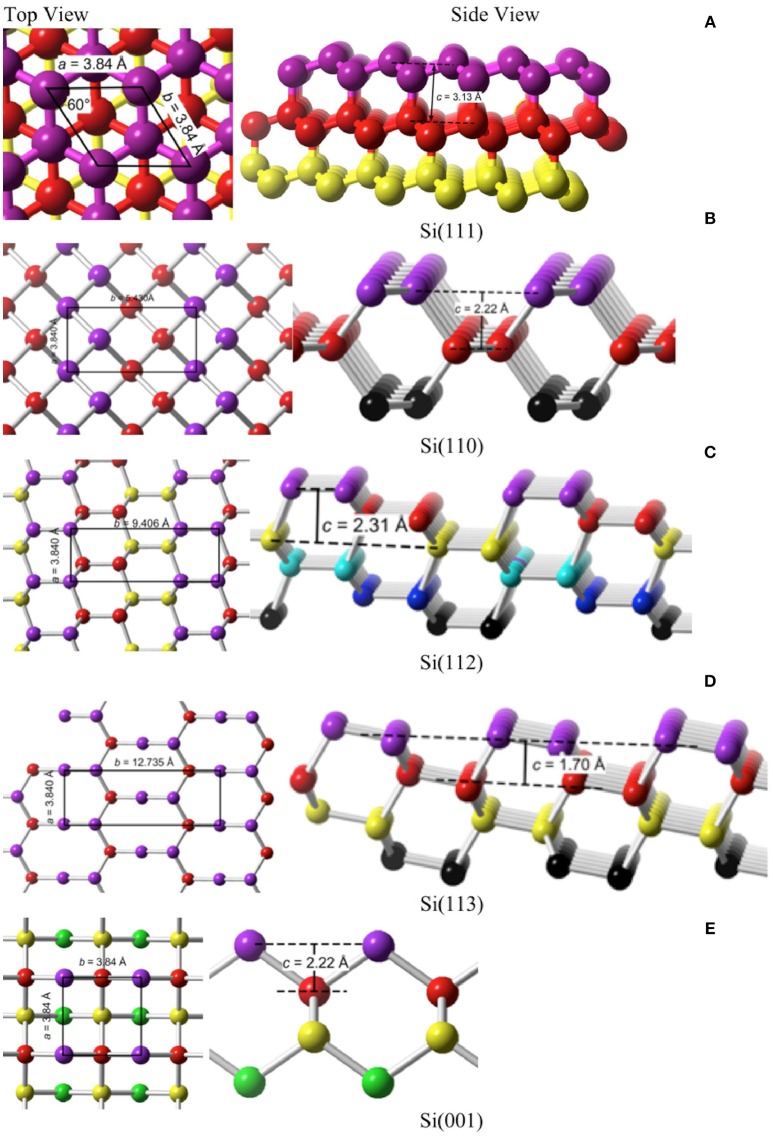
Top and side views of the fives planes that are most likely to be encountered in MACE. **(A)** Si(111), **(B)** Si(110), **(C)** Si(112), **(D)** Si(113), **(E)** Si(001).

The planes most commonly mentioned as relevant to MACE of Si are the {111}, {110}, {112}, {113}, and {001}. The structures of these planes are shown in Figure 6.

A simple model to estimate the surface energy of various Si crystal faces has been described by Dabrowski and Müssig (2000). The cohesive energy of Si is *E*_c_ = 4.63 eV, which is the energy released when a crystal is formed from atoms. Si is tetravalent, that is, each Si atom forms *n*_b_ = 4 bonds and each bond is a two-center bond to which *n*_a_ = 2 atoms contribute. By microscopic reversibility, the energy released in making a bond is equal and opposite to the energy required to break that bond, *E* = *n*_a_*E*_c_/*n*_b_. The surface energy *E*_*hkl*_, i.e., the energy required to form a unit of surface area in the (*hkl*) plane, is estimated by the product of the bond dissociation energy *E* with the areal density of broken bonds. The number of broken bonds *N*_*hkl*_ divided by twice the surface area 2*A*_*hkl*_ (two surfaces are formed from cleaving the crystal) can be calculated from consideration of the unit cell for each plane (*hkl*). The surface energy is, therefore,

(10) $$\begin{array}{r} \gamma_{hkl}=\frac{EN_{hkl}}{2A_{hkl}}=\frac{E_{\text{c}}\;N_{hkl}}{4A_{hkl}} \end{array}$$

Applying Equation (10) we obtain the results in Table 1 for γ_*hkl*_. Si(111) has a rhombohedral unit cell with equal side lengths *a* and apex angle α = 60°; hence, an area of *A* = *a*^2^sinα. All of the other planes have rectangular or square unit cells with side lengths *a* and *b* as indicated in Figure 6 and Table 1. The stability of a clean surface scales as the inverse of the number of broken bonds per unit area. On this basis, the most stable surface is Si(111) and the least is Si(001). Semiconductor surfaces, of course, are known to reconstruct to minimize both the number of dangling bonds and the surface energy. We will not consider reconstructions here because H-termination is able to lift the reconstructions of the bare surface and relax them close to the bulk terminated structure. This is relevant because Si remains H-terminated throughout etching when etching follows the Gerischer mechanism (Kolasinski, 2003).

**Table 1:** The parameters required for the calculation of the surface energy γ_*hkl*_ and etch energy $\gamma_{hkl}^{\text{etch}}$ are collected for the five most commonly encountered planes of Si in MACE.

**Ideal surface**	**Unit cell dimensions Å**	**Cell area *A* Å^**2**^**	**Broken bonds *N* per cell**	**Surface energy ***γ_*hkl*_***eV Å^**−2**^**	**Bonds etched *N*_**e**_ per cell**	**Etch energy $\gamma_{hkl}^{\text{etch}}$eV Å^**−2**^**
Si(111)	*a* = 3.840, α = 60°, *c* = 3.13	12.8	1	0.091	4	0.725
Si(110)	*a* = 3.840, *b* = 5.430, *c* = 2.22	20.9	2	0.111	4	0.444
Si(112)	*a* = 3.840, *b* = 9.406, *c* = 2.31	36.1	4	0.128	9	0.577
Si(113)	*a* = 3.840, *b* = 12.735, *c* = 1.70	48.9	6	0.142	8	0.379
Si(001)	*a* = 3.840, *b* = 3.840, *c* = 2.22	14.7	2	0.157	2	0.314

N is the number of dangling bonds formed per unit cell. The surface unit cell has area A.

*N_e_ is the number of Si–Si bonds broken by etching per unit cell*.

The energy cost per unit area of etching a plane of Si, $\gamma_{hkl}^{\text{etch}}$, is different than the surface energy. It is given by the energy required to break a Si–Si bond times the areal density of Si–Si bonds that must be etched to reveal the next surface unit cell

(11) $$\begin{array}{r} \gamma_{hkl}^{\text{etch}}=\frac{EN_{\text{e},hkl}}{A_{hkl}}=\frac{E_{\text{c}}{\;N}_{\text{e},hkl}}{2A_{hkl}}. \end{array}$$

A total of *N*_e, hkl_ Si–Si bonds per unit cell are dissociated by etching as detailed in Table 1. These values are used to calculate the values of $\gamma_{hkl}^{\text{etch}}$ that appear in the last column of Table 1. $\gamma_{hkl}^{\text{etch}}$ is the energy per unit area required to remove one surface unit cell as atoms, which has a depth *c*_*hkl*_ that depends on the surface crystallography of the etch front. On the {001}, {113}, and {110} surfaces, this corresponds to removing the uppermost layer of surface atoms. On {111} and {112} planes, a bilayer must be removed.

The term $\gamma_{hkl}^{\text{etch}}$ has not been quantified previously. Nonetheless, previous explanations of the crystallographic dependence—sometimes loosely referred to as the back bond model (Peng et al., 2008; Huang et al., 2009, 2010, 2012; Bai et al., 2013; Ouertani et al., 2014; Ghosh and Giri, 2016; Jiang et al., 2016; Jiao et al., 2016; Li et al., 2016)—have attempted to explain the crystallographic dependence of MACE with respect to this one term. *A misinterpretation that frequently appears in connection with the back bond model is that the back bond strength varies with crystallography*. This is false. The back bond strength does not possess a specific crystallographic dependence. Rather, the surface atoms experience different co-ordinations as the result of variations in surface crystallography. Therefore, the energy required to remove a specific surface atom can depend on the surface crystallography as well as on which of its neighbors have been removed previously. As shown by the final column in Table 1, the density of back bonds that must be broken per unit area varies with crystallographic plane. When averaged over the unit cell, an estimate of the energy required to etch the Si surface per unit area is simply the product of the mean bond energy and the number of bonds that have to be broken per unit area.

The term $\gamma_{hkl}^{\text{etch}}$ does not consider that the etched atom is coordinated, e.g., first as HSiF_3_, as it leaves the surface rather than as a Si atom. This will change the absolute energetics; however, all Si surfaces have the same etch product and, therefore, the relative etch energy will not be affected by the chemical nature of the etch product. Note also that $\gamma_{hkl}^{\text{etch}}$ is a thermodynamic rather than a kinetic parameter, i.e., it is not an activation energy but the difference in energy per unit area between the initial and final states. The expression does not take steric factors into account, which influence the kinetics but not thermodynamics of pore formation. The formation of a 5-fold transition state is commonly invoked to explain the crystallographic dependence of alkaline etching of Si (Hines et al., 1994; Baum and Schiffrin, 1998). An implicit assumption of the back bond model is that *E*_a, etch_ scales with the value of $\gamma_{hkl}^{\text{etch}}$, in other words, that the activation energy follows a linear Brønsted-Evans-Polanyi relation as is often found in catalysis (Bligaard et al., 2004). Our experimentally measured values for the etching activation energy (0.34–0.44 eV) are comparable to the Si(001) value of $\gamma_{hkl}^{\text{etch}}$ reported in Table 1 (0.31 eV), which lends credence to the model presented here.

Even without consideration of the effects of adsorbates or the final chemical form of the etch product, the term $\gamma_{hkl}^{\text{etch}}$ alone does not fully describe the energy required to form a pore or SiNW by etching. Etching one layer of Si atoms to reveal a new surface unit cell requires etching a depth *c*_*hkl*_, that depends on the surface crystallography as shown in Figure 6. Thus, one must also consider the energy required to create the sidewalls of the pore/SiNW. It is important to recognize that etching is not an equilibrium process that is creating structures with the lowest possible surface energy. Rather, it is a kinetically-controlled process that is influenced by the trajectory that removes atoms along the minimum energy path.

**Figure 7:**
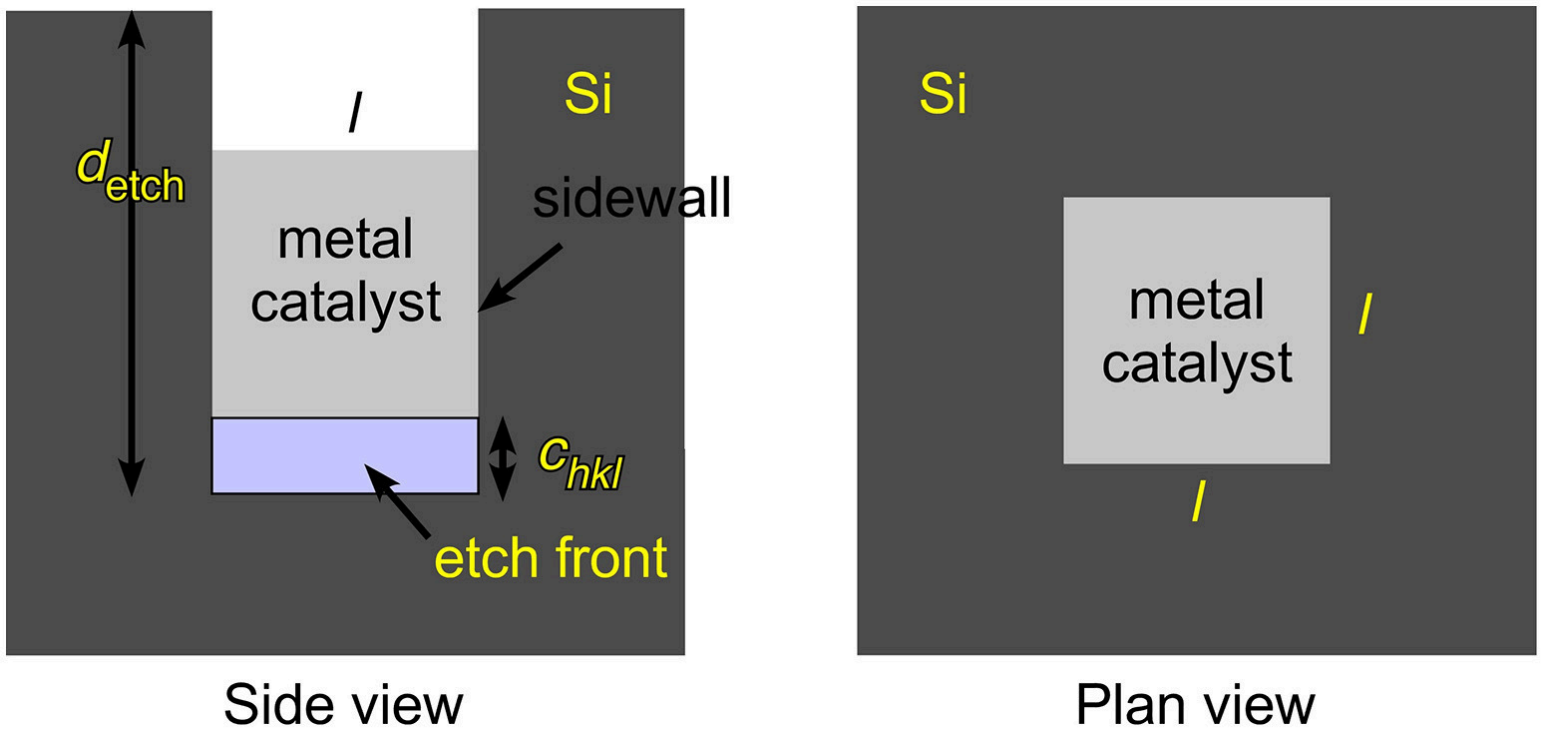
Metal assisted catalyzed etching (MACE) is represented schematically for a cubic metal catalyst of side length *l* etching with a planar etch front that is perpendicular to the sidewalls. The sidewalls need not be perpendicular to the macroscopic normal to the wafer/particle that is being etched. The total etch depth *d*_etch_ is obtained by stepwise removal of one surface unit cell after another with a step depth *c*_hkl_ that depends on the surface crystallography of the plane that contains the etch front.

We consider one metal catalyst particle, square in cross section with side length *l*, and the formation of one rectangular pore etched to a depth *d*_etch_ beneath it. We conceptualize etching as a stepwise process in which the depth of one surface unit cell *c*_*hkl*_ at a time is removed. We seek to derive an expression for the energy required to remove one such surface unit cell layer. The expression (assuming etching in only one direction and sidewalls of only one crystallographic orientation with sidewalls perpendicular to a planar etch front, as shown in Figure 7) is

(12) $$\begin{array}{r} E_{\text{pore}}=\gamma_{hkl}^{\text{etch}}A_{\text{cat}}+{\gamma^{\prime }}_{hkl}^{\text{etch}}A_{\text{sw}} \end{array}$$

where *A*_cat_ is the area beneath the catalyst

(13) $$\begin{array}{r} A_{\text{cat}}=l^{2}={\left(\eta_{1}\;a_{\text{Si}}\right)}^{2} \end{array}$$

which is expressed using the ratio of the length to the Si lattice constant η_1_ = *l*/*a*_Si_ and *A*_sw_ is the sidewall area

(14) $$\begin{array}{r} A_{\text{SW}}=4lc_{hkl}=4\eta_{1}\;\eta_{2}\;a_{\text{Si}}^{2} \end{array}$$

expressed in terms of the ratio η_2_ = *c*_*hkl*_/*a*_Si_. The Si lattice constant is *a*_Si_ = 5.43095 Å (Sze and Ng, 2006).

The prime in Equation (12) indicates that the sidewalls will, in general, have a different crystallographic orientation than the etch front. The total energy to create a pore of some macroscopic depth *d*_etch_ would then be the sum of Equation (12) over the number of steps *n*_s_ required to etch to this depth, *d*_etch_ = *n*_s_*c*_*hkl*_, that is, *E*_total_ = *n*_s_*E*_pore_. Should etching reveal multiple sidewall orientations or the direction of etching change during etching, e.g., to form zigzag SiNW, then appropriate summations over surface energies and areas would have to be taken.

Substituting for the areas, the energy required to perform one step of pore formation is

(15) $$\begin{array}{r} E_{\text{pore}}=\eta_{1}\;a_{\text{Si}}^{2}\left[\eta_{1}\;\gamma_{hkl}^{\text{etch}}+4\eta_{2}\;{\gamma^{\prime }}_{hkl}^{\text{etch}}\right]. \end{array}$$

The importance of the etch-front term $\eta_{1}\;\gamma_{hkl}^{\text{etch}}$compared to the sidewall term $4\eta_{2}\;{\gamma^{\prime }}_{hkl}^{\text{etch}}$ is determined by the ratio η_2_/η_1_, which in turn is determined by the lateral size of the catalyst. For *l* > 15 nm, the sidewall term is < 10% as large as the etch-front term, and can then be neglected. Only for small nanoparticle catalysts with *l*<15 nm must the sidewall term be considered.

At this point it is interesting to compare to the “inverse problem,” that is, to the formation of SiNWs by growth rather than etching. Wu et al. (2004) have shown that SiNWs grown with a Au catalyst in a vapor-liquid-solid (VLS) process grow along the 〈111〉 direction for diameters above 20 nm, in the 〈112〉 direction for diameters between 10 and 20 nm, and along the 〈110〉 direction for diameters between 3 and 10 nm. They suggested that at large diameters, formation of the lowest surface energy plane, i.e., the (111) plane, in the growth front dominates the energetics. However, for the smallest diameters, sidewall energy becomes the dominant factor and leads to 〈110〉-directed growth. For the intermediate region, 〈112〉-directed growth occurs because the (112) plane, which can be thought of as a stepped surface intermediate between (111) and (110) planes, results from a balance between the growth front and sidewall terms. This line of reasoning is consistent with the etch model developed here and the results of Equation (15).

**Figure 8:**
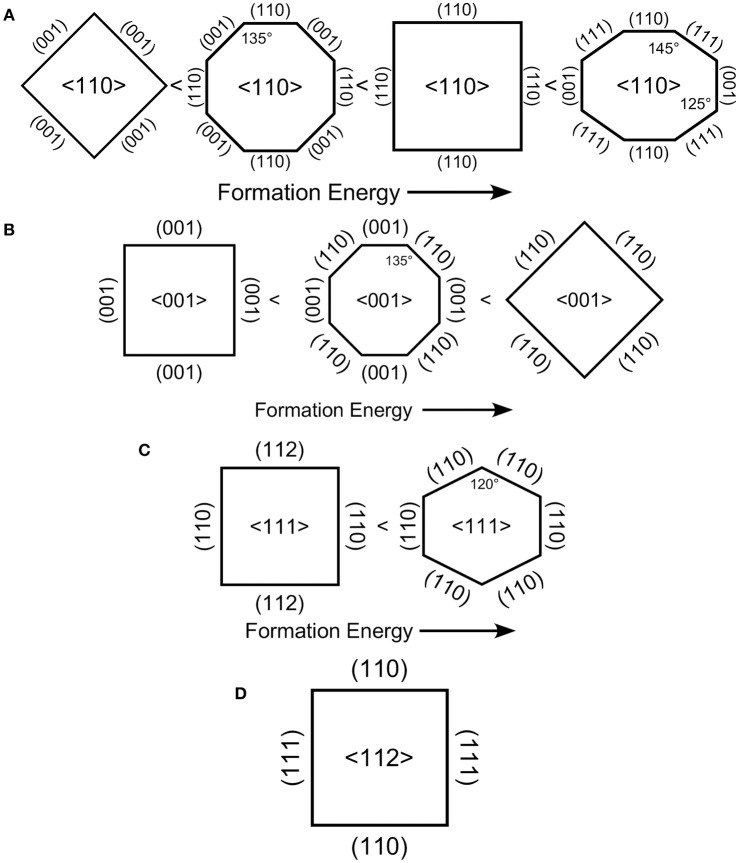
Viewed down the long axis of a vertical pore (or pillar), the crystallography of the sidewalls is shown. For four directions **(A)** 〈110〉, **(B)** 〈001〉, **(C)** 〈111〉, **(D)** 〈112〉, vertical pores can be formed. Depending on the crystallography of the sidewall terminations, these ideal pores would exhibit facets that form either rectangular, hexagonal or octagonal cross-sections. A vertical pore in the direction is not possible as only two planes make right angles with it.

However, even if on energetic grounds the sidewall term can be neglected compared to the etch-front term, the influence of the sidewalls cannot be ignored. The crystallographic specificity of MACE is determined not only by the energy required to advance the etch front, but also by the combinations of planes that are allowed by crystallography. The sidewall term is always lowest for 〈001〉- and 〈113〉-directed facets. However, these cannot always be formed. The allowed combinations for sidewall facets that are perpendicular to the pore/NW axis are shown in Figure 8. First note that 〈113〉-directed pillars cannot form pores/NW with all sidewalls perpendicular to the long axis. This is because only two {110}-directed facets are perpendicular to the 〈113〉-direction. The lowest energy sidewalls for 〈113〉-directed pores/NW are {111}-directed facets but these are inclined to the 〈113〉 direction by 71°. That Peng et al. (2008) did not observed 〈113〉-directed pores when etching Si(113) substrates demonstrates that there are limits to the inclination of the sidewalls.

The results in Table 1 show that the thermodynamic stability follows the order Si(001) < Si(113) < Si(112) < Si(110) < Si(111). However, the susceptibility to etching follows the order Si(001) > Si(113) > Si(110) > Si(112) > Si(111). Simple application of the back bond model predicts that 〈001〉-directed pores/SiNW would be the most commonly observed, which is confirmed experimentally, and 〈111〉-directed pores/SiNW should not be observed. 〈111〉-directed pores/SiNW are observed when a perforated metal film is used as a catalyst, which demonstrates that a highly-correlated motion of metal catalyst is required to overcome the energetics of the $\gamma_{hkl}^{\text{etch}}$ term. Obviously, interpretation of the back bond model in terms only of the energy required to etch in a given crystallographic direction is overly simplistic as it does not include correlation between catalyst particles nor the dependence on the size of the catalyst particle. The above analysis does, however, show us that preferences for sidewall orientation are determined by the lowest barrier to etching rather than by selection of the most stable surface plane.

By combining the results in Table 1 and Figure 8, we can determine the most likely sidewall terminations for each etching direction. Ideally 〈112〉-directed etching is predicted to form square or rectangular pores/NW with {111}- and {110}-oriented facets.

Ideally, 〈111〉-directed etching is predicted to form either hexagonal or rectangular pores/NW. However, the hexagonal pores/NW with {110}-directed facets should have substantially lower formation energy than rectangular pores/NW that exhibit both {110}- and {111}-directed facets.

Similarly, 〈001〉-directed etching is predicted to form either rectangular or octagonal pores/NW. Rectangular pores/NW with {001}-directed facets should have substantially lower formation energy than rectangular pores/NW that exhibit {110}-directed facets. The octagonal structure with a combination of both {110}- and {001}-directed facets has a formation energy averaged between these two.

Finally, 〈110〉-directed etching exhibits the richest variety of pore/NW structure. {111}-directed facets have higher formation energy than {110}-directed facets, which are higher than {100}-directed facets. The {111} facets can only form a closed structure with both {100} and {111} facets to form an irregular octagon as the highest energy structure. Next is a rectangular pore/NW with {110} facets followed by an octagonal structure with a combination of both {110}- and {100}-directed facets. Rectangular pores/NW that exhibit {100}-directed facets have by far the lowest formation energy.

Strong evidence for a porous region along the etch front has been presented. Geyer et al. (2012) reported extensive experiments and high-resolution TEM images that reveal a porous Si region beneath a patterned Ag film etched with a solution consisting of 5.65 M HF and 0.10 M H_2_O_2_. Lai et al. (2013) fitted extensive kinetic data to a cyclical process involving the formation of a porous etch front using Au catalyst and H_2_O_2_ + HF. Chourou et al. (2010) found a porous region subsequent to metal-assisted etching under anodic polarization.

Contradictory reports also exist. TEM has been interpreted as consistent with a solid etch front (Huang et al., 2010) for 〈111〉-oriented SiNW etched at high H_2_O_2_ concentrations with Ag particles. A solid etch front was reported (Liu et al., 2013) for etching catalyzed by Au nanoparticles in H_2_O_2_ + HF though it should be noted that the etched features were consistently larger than the nanoparticles and that a thin porous layer could easily have been removed by the aqua regia used to dissolve the Au before microscopy was performed.

## Conclusion

Metal-assisted catalytic etching (MACE) performed with Ag nanoparticles as catalysts and H_2_O_2_ as oxidant efficiently porosifies single-crystal Si wafers, single-crystal electronics grade Si powders and polycrystalline metallurgical grade silicon powders. Etching with 0.15 M H_2_O_2_ is found to preferentially proceed in a 〈001〉 direction with an activation energy of ~0.4 eV on substrates with (001), (110), and (111) orientations. A quantitative model based on the energy required to remove a surface unit cell through etching explains the preference for etching in the 〈001〉 directions, and is consistent with the measured activation energies. This model also predicts that catalyst particle size may influence the energetics of etch track pore formation. Etching of metallurgical grade powders produces particles covered primarily with porous silicon in the form of interconnected ridges. Capillary forces and ultrasonic agitation can be used to pulverize the porous layer to form silicon nanowires and bundles of nanowires. Strongly attractive electrostatic and van der Waals interactions adhere the nanoparticle catalyst to the Si surface ensuring that the nanoparticles remain in intimate contact with the Si surface throughout the etch process regardless of whether etching occurs on a wafer or on a powder particle. The attractive forces explain why powder particles are etched equivalently on all exposed faces of powder particles because they draw the catalyst toward the interior of the particle irrespective of which direction is vertical.

## Author Contributions

KK was primarily responsible for the design of etching experiments, the development of the model, calculations and the writing of the first draft of the manuscript. BU performed the etching experiments and preliminary SEM characterization. MA designed the high-resolution microscopy characterization, which was performed by AE.

### Conflict of Interest Statement

Independently of the work presented in the present publication, one of the authors (KK) has provided expert advice to OneD Material LLC during proceedings related to patents held by Nexeon Ltd. The remaining authors declare that the research was conducted in the absence of any commercial or financial relationships that could be construed as a potential conflict of interest.

## References

[B1] Anglin E. J. Cheng L. Freeman W. R. Sailor M. J. (2008). Porous silicon in drug delivery devices and materials. Adv. Drug Deliver. Rev. 60, 1266–1277. 10.1016/j.addr.2008.03.017 18508154 PMC2710886

[B2] Aricò A. S. Bruce P. Scrosati B. Tarascon J. M. Van Schalkwijk W. (2005). Nanostructured materials for advanced energy conversion and storage devices. Nat. Mater. 4, 366–377. 10.1038/nmat1368 15867920

[B3] Armstrong M. J. O'dwyer C. Macklin W. J. Holmes J. D. (2014). Evaluating the performance of nanostructured materials as lithium-ion battery electrodes. Nano Res. 7, 1–62. 10.1007/s12274-013-0375-x

[B4] Ashley C. E. Carnes E. C. Phillips G. K. Padilla D. Durfee P. N. Brown P. A.. (2011). The targeted delivery of multicomponent cargos to cancer cells by nanoporous particle-supported lipid bilayers. Nat. Mater. 10, 389–397. 10.1038/nmat2992 21499315 PMC3287066

[B5] Bai F. To W. K. Huang Z. (2013). Porosification-induced back-bond weakening in chemical etching of n-Si(111). J. Phys. Chem. C 117, 2203–2209. 10.1021/jp311999u

[B6] Baum T. Schiffrin D. J. (1998). Mechanistic aspects of anisotropic dissolution of materials etching of single-crystal silicon in alkaline solutions. J. Chem. Soc. Faraday Trans. 94, 691–694. 10.1039/a707473e

[B7] Bellet D. Canham L. (1998). Controlled drying: the key to better quality porous semiconductors. Adv. Mater. 10, 487–490. 10.1002/(SICI)1521-4095(199804)10:6<487_H::AID-ADMA487>3.0.CO;2-T 21647985

[B8] Bligaard T. Nørskov J. K. Dahl S. Matthiesen J. Christensen C. H. Sehested J. (2004). The brønsted-evans-polanyi relation and the volcano curve in heterogeneous catalysis. J. Catal. 224, 206–217. 10.1016/j.jcat.2004.02.034

[B9] Blomgren G. E. (2017). The development and future of lithium ion batteries. J. Electrochem. Soc. 164, A5019–A5025. 10.1149/2.0251701jes

[B10] Bruce P. G. Scrosati B. Tarascon J. M. (2008). Nanomaterials for rechargeable lithium batteries. Angew. Chem. Int. Ed. Engl. 47, 2930–2946. 10.1002/anie.200702505 18338357

[B11] Campbell S. D. Jones L. A. Nakamichi E. Wei F. X. Zajchowski L. D. Thomas D. F. (1995). Spectral and structural features of porous silicon prepared by chemical and electrochemical etching processes. J. Vac. Sci. Technol. B 13, 1184–1189. 10.1116/1.588233

[B12] Chan C. K. Peng H. Liu G. Mcilwrath K. Zhang X. F. Huggins R. A.. (2008). High-performance lithium battery anodes using silicon nanowires. Nat. Nanotech. 3, 31–35. 10.1038/nnano.2007.411 18654447

[B13] Chartier C. Bastide S. Levy-Clement C. (2008). Metal-assisted chemical etching of silicon in HF-H_2_O_2_. Electrochim. Acta 53, 5509–5516. 10.1016/j.electacta.2008.03.009

[B14] Cheng S. L. Chung C. H. Lee H. C. (2008). A study of the synthesis, characterization, and kinetics of vertical silicon nanowire arrays on (001)Si substrates. J. Electrochem. Soc. 155, D711–D714. 10.1149/1.2977548

[B15] Chiappini C. Liu X. Fakhoury J. R. Ferrari M. (2010). Biodegradable porous silicon barcode nanowires with defined geometry. Adv. Func. Mater. 20, 2231–2239. 10.1002/adfm.201000360 21057669 PMC2971684

[B16] Chourou M. L. Fukami K. Sakka T. Virtanen S. Ogata Y. H. (2010). Metal-assisted etching of p-type silicon under anodic polarization in HF solution with and without H_2_O_2_. Electrochim. Acta 55, 903–912. 10.1016/j.electacta.2009.09.048

[B17] Dabrowski J. Müssig H. J. (2000). Silicon surfaces and formation of interfaces: basic science in the industrial world. Singapore; River Edge, NJ: World Scientific, 47–50.

[B18] Dudley M. E. Kolasinski K. W. (2008). Wet etching of pillar covered silicon surface: formation of crystallographically defined macropores. J. Electrochem. Soc. 155, H164–H171. 10.1149/1.2826292

[B19] Föll H. Christophersen M. Carstensen J. Hasse G. (2002). Formation and application of porous silicon. Mater. Sci. Eng. R 39, 93–141. 10.1016/S0927-796X(02)00090-6

[B20] Geyer N. Fuhrmann B. Huang Z. P. De Boor J. Leipner H. S. Werner P. (2012). Model for the mass transport during metal-assisted chemical etching with contiguous metal films as catalysts. J. Phys. Chem. C 116, 13446–13451. 10.1021/jp3034227

[B21] Ghosh R. Giri P. K. (2016). Efficient visible light photocatalysis and tunable photoluminescence from orientation controlled mesoporous Si nanowires. RSC Adv. 6, 35365–35377. 10.1039/C6RA05339D

[B22] Griffiths D. J. (1981). Introductions to Electrodynamics, Englewood Cliffs. New Jersey, NJ: Prentice-Hall.

[B23] Han H. Huang Z. P. Lee W. (2014). Metal-assisted chemical etching of silicon and nanotechnology applications. Nano Today 9, 271–304. 10.1016/j.nantod.2014.04.013

[B24] Heinrich J. L. Curtis C. L. Credo G. M. Kavanagh K. L. Sailor M. J. (1992). Luminescent colloidal silicon suspensions from porous silicon. Science 255:66. 10.1126/science.255.5040.66 17739915

[B25] Hildreth O. J. Brown D. Wong C. P. (2011). 3D Out-of-plane rotational etching with pinned catalysts in metal-assisted chemical etching of silicon. Adv. Func. Mater. 21, 3119–3128. 10.1002/adfm.201100279

[B26] Hildreth O. J. Rykaczewski K. Fedorov A. G. Wong C. P. (2013). A DLVO model for catalyst motion in metal-assisted chemical etching based upon controlled out-of-plane rotational etching and force-displacement measurements. Nanoscale 5, 961–970. 10.1039/C2NR32293E 23238167

[B27] Hines M. A. Chabal Y. J. Harris T. D. Harris A. L. (1994). Measuring the structure of etched silicon surfaces with Raman spectroscopy. J. Chem. Phys. 101, 8055–8072. 10.1063/1.468232

[B28] Hochbaum A. I. Yang P. (2010). Semiconductor nanowires for energy conversion. Chem. Rev. 110, 527–546. 10.1021/cr900075v 19817361

[B29] Huang Z. Shimizu T. Senz S. Zhang Z. Zhang X. Lee W.. (2009). Ordered arrays of vertically aligned [110] silicon nanowires by suppressing the crystallographically preferred etching directions. Nano Lett. 9, 2519–2525. 10.1021/nl803558n 19480399

[B30] Huang Z. Wang R. Jia D. Maoying L. Humphrey M. G. Zhang C. (2012). Low-cost, large-scale, and facile production of Si nanowires exhibiting enhanced third-order optical nonlinearity. ACS Appl. Mater. Interfaces 4, 1553–1559. 10.1021/am201758z 22329903

[B31] Huang Z. P. Shimizu T. Senz S. Zhang Z. Geyer N. Gösele U. (2010). Oxidation rate effect on the direction of metal-assisted chemical and electrochemical etching of silicon. J. Phys. Chem. C 114, 10683–10690. 10.1021/jp911121q

[B32] Israelachvili J. N. (2011). Intermolecular and Surface Forces. Burlington, MA, Academic Press.

[B33] Jiang B. Li M. Liang Y. Bai Y. Song D. Li Y.. (2016). Etching anisotropy mechanisms lead to morphology-controlled silicon nanoporous structures by metal assisted chemical etching. Nanoscale 8, 3085–3092. 10.1039/C5NR07327H 26785718

[B34] Jiao X. Chao Y. Wu L. Yao A. (2016). Metal-assisted chemical etching of silicon 3D nanostructure using direct-alternating electric field. J. Mater. Sci. 27, 1881–1887. 10.1007/s10854-015-3968-1

[B35] Kamat P. V. (2007). Meeting the clean energy demand: nanostructure architectures for solar energy conversion. J. Phys. Chem. C 111, 2834–2860. 10.1021/jp066952u

[B36] Kang D. K. Corno J. A. Gole J. L. Shin H. C. (2008). Microstructured nanopore-walled porous silicon as an anode material for rechargeable lithium batteries. J. Electrochem. Soc. 155, A276–A281. 10.1149/1.2836570

[B37] Kasavajjula U. Wang C. Appleby A. J. (2007). Nano- and bulk-silicon-based insertion anodes for lithium-ion secondary cells. J. Power Sour. 163, 1003–1039. 10.1016/j.jpowsour.2006.09.084

[B38] Kaukonen A. M. Laitinen L. Salonen J. Tuura J. Heikkilä T. Limnell T.. (2007). Enhanced *in vitro* permeation of furosemide loaded into thermally carbonized mesoporous silicon (TCPSi) microparticles. Euro. J. Pharm. Biopharm. 66, 348–356. 10.1016/j.ejpb.2006.11.021 17240128

[B39] Kilpeläinen M. Riikonen J. Vlasova M. A. Huotari A. Lehto V. P. Salonen J.. (2009). *In vivo* delivery of a peptide, ghrelin antagonist, with mesoporous silicon microparticles. J. Control. Release 137, 166–170. 10.1016/j.jconrel.2009.03.017 19345247

[B40] Kim H. Han B. Choo J. Cho J. (2008). Three-dimensional porous silicon particles for use in high-performance lithium secondary batteries. Angew. Chem. Int. Ed. Engl. 47, 10151–10154. 10.1002/anie.200804355 19016293

[B41] Kolasinski K. W. (2003). The mechanism of Si etching in fluoride solutions. Phys. Chem. Chem. Phys. 5, 1270–1278. 10.1039/b212108e

[B42] Kolasinski K. W. (2014). The mechanism of galvanic/metal-assisted etching of silicon. Nanoscale Res. Lett. 9:432. 10.1186/1556-276X-9-432 25221459 PMC4149979

[B43] Kolasinski K. W. (2016). Electron transfer during metal-assisted and stain etching of silicon. Semicond. Sci. Technol. 31:014002 10.1088/0268-1242/31/1/014002

[B44] Kolasinski K. W. Barclay W. B. (2013). Stain etching of silicon with and without the aid of metal catalysts. ECS Trans. 50, 25–30. 10.1149/05037.0025ecst

[B45] Kolasinski K. W. Barclay W. B. Sun Y. Aindow M. (2015). The stoichiometry of metal assisted etching of Si in V_2_O_5_ + HF and HOOH + HF solutions. Electrochim. Acta 158, 219–228. 10.1016/j.electacta.2015.01.162

[B46] Kolasinski K. W. Gimbar N. J. Yu H. Aindow M. Mäkilä E. Salonen J. (2017). Regenerative electroless etching of silicon. Angew. Chem. 55, 624–627. 10.1002/anie.201610162 27925365

[B47] Kolasinski K. W. Unger B. A. Yu H. Ernst A. T. Aindow M. Mäkilä E. (2018). Hierarchical porous silicon and porous silicon nanowires produced with regenerative electroless etching (ReEtching) and metal assisted catalytic etching (MACE). ECS Trans. 86, 65–70. 10.1149/08601.0065ecst

[B48] Lai C. Q. Cheng H. Choi W. K. Thompson C. V. (2013). Mechanics of catalyst motion during metal assisted chemical etching of silicon. J. Phys. Chem. C 117, 20802–20809. 10.1021/jp407561k

[B49] Lee J. K. Oh C. Kim N. Hwang J. Y. Sun Y. K. (2016). Rational design of silicon-based composites for high-energy storage devices. J. Mater. Chem. A 4, 5366–5384. 10.1039/C6TA00265J

[B50] Lee S. W. Mcdowell M. T. Choi J. W. Cui Y. (2011). Anomalous shape changes of silicon nanopillars by electrochemical lithiation. Nano Lett. 11, 3034–3039. 10.1021/nl201787r 21657250

[B51] Leisner M. Cojocaru A. Ossei-Wusu E. Carstensen J. Föll H. (2010). New applications of electrochemically produced porous semiconductors and nanowire arrays. Nanoscale Res. Lett. 5, 1502–1506. 10.1007/s11671-010-9669-z 20730118 PMC2920402

[B52] Li M. Li Y. Liu W. Yue L. Li R. Luo Y. (2016). Metal-assisted chemical etching for designable monocrystalline silicon nanostructure. Mater. Res. Bull. 76, 436–449. 10.1016/j.materresbull.2016.01.006

[B53] Li X. L. (2012). Metal assisted chemical etching for high aspect ratio nanostructures: a review of characteristics and applications in photovoltaics. Curr. Opin. Solid State Mater. Sci. 16, 71–81. 10.1016/j.cossms.2011.11.002

[B54] Liu G. Young K. L. Liao X. Personick M. L. Mirkin C. A. (2013). Anisotropic nanoparticles as shape-directing catalysts for the chemical etching of silicon. J. Am. Chem. Soc. 135, 12196–12199. 10.1021/ja4061867 23905761

[B55] Liu X. H. Zhong L. Huang S. Mao S. X. Zhu T. Huang J. Y. (2012). Size-dependent fracture of silicon nanoparticles during lithiation. ACS Nano 6, 1522–1531. 10.1021/nn204476h 22217200

[B56] Ma J. Wen L. Dong Z. Zhang T. Wang S. Jiang L. (2013). Aligned silicon nanowires with fine-tunable tilting angles by metal-assisted chemical etching on off-cut wafers. Rapid Res. Lett. 7, 655–658. 10.1002/pssr.201307190

[B57] Mai L. Tian X. Xu X. Chang L. Xu L. (2014). Nanowire electrodes for electrochemical energy storage devices. Chem. Rev. 114, 11828–11862. 10.1021/cr500177a 25290387

[B58] McSweeney W. Geaney H. O'Dwyer C. (2015). Metal-assisted chemical etching of silicon and the behavior of nanoscale silicon materials as Li-ion battery anodes. Nano Res. 8, 1395–1442. 10.1007/s12274-014-0659-9

[B59] Micheli L. Sarmah N. Luo X. Reddy K. S. Mallick T. K. (2013). Opportunities and challenges in micro- and nano-technologies for concentrating photovoltaic cooling: a review. Renew. Sustain. Energy Rev. 20, 595–610. 10.1016/j.rser.2012.11.051

[B60] Mills D. Kolasinski K. W. (2005). A Non-lithographic method to form ordered arrays of silicon pillars and macropores. J. Phys. D 38, 632–636. 10.1088/0022-3727/38/4/017

[B61] Mills D. Nahidi M. Kolasinski K. W. (2005). Stain etching of silicon pillars and macropores. Phys. Status Solidi A 202, 1422–1426. 10.1002/pssa.200461119

[B62] Ouertani R. Hamdi A. Amri C. Khalifa M. Ezzaouia H. (2014). Formation of silicon nanowire packed films from metallurgical-grade silicon powder using a two-step metal-assisted chemical etching method. Nanoscale Res. Lett. 9:574. 10.1186/1556-276X-9-574 25349554 PMC4209156

[B63] Pei Z. Hu H. Li S. Ye C. (2017). Fabrication of orientation-tunable si nanowires on silicon pyramids with omnidirectional light absorption. Langmuir 33, 3569–3575. 10.1021/acs.langmuir.6b04068 28368596

[B64] Peng K. Wu Y. Fang H. Zhong X. Xu Y. Zhu J. (2005). Uniform, axial-orientation alignment of one-dimensional single-crystal silicon nanostructure arrays. Angew. Chem. 44, 2737–2742. 10.1002/anie.200462995 15812791

[B65] Peng K. Q. Lu A. J. Zhang R. Q. Lee S. T. (2008). Motility of metal nanoparticles in silicon and induced anisotropic silicon etching. Adv. Func. Mater. 18, 3026–3035. 10.1002/adfm.200800371

[B66] Peng K. Q. Zhang M. L. Lu A. J. Wong N. B. Zhang R. Q. Lee S. T. (2007). Ordered silicon nanowire arrays via nanosphere lithography and metal-induced etching. Appl. Phys. Lett. 90:163123 10.1063/1.2724897

[B67] Rezvani S. J. Gunnella R. Neilson D. Boarino L. Croin L. Aprile G.. (2016). Effect of carrier tunneling on the structure of Si nanowires fabricated by metal assisted etching. Nanotechnology 27:345301. 10.1088/0957-4484/27/34/345301 27420163

[B68] Rykaczewski K. Hildreth O. J. Wong C. P. Fedorov A. G. Scott J. H. (2011). Guided three-dimensional catalyst folding during metal-assisted chemical etching of silicon. Nano Lett. 11, 2369–2374. 10.1021/nl200715m 21526791

[B69] Salonen J. Kaukonen A. M. Hirvonen J. Lehto V. P. (2008). Mesoporous silicon in drug delivery applications. J. Pharm. Sci. 97, 632–653. 10.1002/jps.20999 17546667

[B70] Santos H. A. Bimbo L. M. Lehto V. P. Airaksinen A. J. Salonen J. Hirvonen J. (2011). Multifunctional porous silicon for therapeutic drug delivery and imaging. Curr. Drug Discov. Tech. 8, 228–249. 10.2174/157016311796799053 21291407

[B71] Santos H. A. Hirvonen J. (2012). Nanostructured porous silicon materials: potential candidates for improving drug delivery. Nanomedicine 7, 1281–1284. 10.2217/nnm.12.106 22994953

[B72] Shin H. C. Corno J. A. Gole J. L. Liu M. L. (2005). Porous silicon negative electrodes for rechargeable lithium batteries. J. Power Sour. 139, 314–320. 10.1016/j.jpowsour.2004.06.073

[B73] Sze S. M. Ng K. K. (2006). Physics of Semiconductor Devices. New York, NY: Wiley-Interscience.

[B74] Toor F. Miller J. B. Davidson L. M. Duan W. Jura M. P. Yim J.. (2016a). Metal assisted catalyzed etched (MACE) black Si: optics and device physics. Nanoscale 8, 15448–15466. 10.1039/C6NR04506E 27533490

[B75] Toor F. Miller J. B. Davidson L. M. Nichols L. Duan W. Jura M. P.. (2016b). Nanostructured silicon via metal assisted catalyzed etch (MACE): chemistry fundamentals and pattern engineering. Nanotechnology 27:412003. 10.1088/0957-4484/27/41/412003 27609489

[B76] Tsujino K. Matsumura M. (2005). Helical nanoholes bored in silicon by wet chemical etching using platinum nanoparticles as catalyst. Electrochem. Solid State Lett. 8, C193–C195. 10.1149/1.2109347

[B77] Wareing N. Szymanski K. Akkaraju G. R. Loni A. Canham L. T. Gonzalez-Rodriguez R. (2017). *In-vitro* gene delivery with large porous silicon nanocrystals fabricated using cost effective metal-assisted etching. Small 13:1602739 10.1002/smll.201602739 28084695

[B78] Wu Y. Cui Y. Huynh L. Barrelet C. J. Bell D. C. Lieber C. M. (2004). Controlled growth and structures of molecular-scale silicon nanowires. Nano Lett. 4, 433–436. 10.1021/nl035162i

